# Culture, Ageing and the Construction of Pain

**DOI:** 10.3390/geriatrics3030040

**Published:** 2018-07-09

**Authors:** Pauline Lane, David Smith

**Affiliations:** Faculty of Health Social Care and Education, Anglia Ruskin University, Bishop Hall Lane, Chelmsford, Essex, CM1 1SQ, UK

**Keywords:** culture, ageing, pain, idioms of distress, somatisation, cultural concepts of distress, stoicism

## Abstract

In this paper, the authors seek to discuss some of the complexities involved in cross-cultural working in relation to the communication and management of pain in older people. Specifically, the paper addresses the culture construction of ageing and how pain is often constructed as a natural part of ageing. The authors also suggest that with the rise of the ideology of active-ageing, many older people who are disabled or living in chronic pain, may feel a moral imperative to hide pain and ill-health. The discussion extends into looking at the impact of culture and the communication of pain, including specific idioms of distress, somaticize and the lay-management of pain through stoicism. The literature utilised in this paper was based on a thematic review, exploring the cultural dimensions of health, illness and pain in old age. The review also drew on the authors’ previous publications, as well as their extensive community research experience working with ethnic minority communities.

## 1. Introduction

The last national census has highlighted how we are now living in an ‘ageing society’ with an estimated 18% of the population of England and Wales aged 65 years old and above and it is also notable, that we are living in a more multi-cultural society, with the number of Black, Asian and other ethnic minority (BAME) people now accounting for 14% of the total population in England and Wales [1]. Inevitably, this population is also ageing and while currently the BAME population tends to be younger, it has been estimated that the number of people from ethnic minority communities aged 50 and above, will constitute between 22% and 30% of the general population of the UK by 2051 [2,3] and in the future, health and social care practitioners will be working with an increasing number of older people from more ethnically diverse communities. Therefore, an understanding of the role of culture in pain in old age, and the significance of transcultural working, may help practitioners to promote more effective health care and support.

The paper is divided into two sections; initially, the authors briefly examine the cultural construction of ageing and how this can influence older persons’ expectations of pain as they age. The authors also highlight how the social determinants of health over a lifetime, can inform the quality of life of individuals as they age.

The second section of the paper highlights some of the many trans-cultural challenges that health practitioners may face in the clinical setting, when discussing pain with older people who come from a different culture to their own. This section examines working with different idioms of distress, somatisation and cultural concepts of distress.

In the conclusion, the authors consider critiques regarding the causative role of culture that tend to focus on the relation between social structure and culture; and the political and policy implications that cultural perspectives can engender. They argue that to avoid ‘cultural determinism’ a nuanced approach to understanding the role of culture in the expression and management of pain encompassing its dynamic, generational and contextual aspects is required.

The literature utilised in this paper was based on a thematic review, exploring the cultural dimensions of health, illness and pain in old age. The review also drew on the authors’ previous publications, as well as their extensive community research experience working with ethnic minority communities.

## 2. The Cultural Construction of Ageing

While we all get old, people’s experiences of ageing is mediated by a number of intersecting factors including the cultural construction of ageing. This has implications for the study of older people and pain management, because in many cultures, pain is often considered to be a ‘natural’ part of the ageing process.

### 2.1. Thinking about ‘Culture’

Everyone lives in a culture and while it has been variously defined, it is often understood to be the traits, beliefs and practices acquired through primary and secondary socialisation that provides the background or context of our lives and shapes how we understand and give meaning to our lives [4]. For example, our social class, ethnicity, gender, religion, language, family and education are all shaped by the cultural and historical context of our lives. Yet, as Handler suggests, culture needs to be understood as a dynamic process as it “gets constructed, deconstructed, and reconstructed” [5].

### 2.2. Culture and the Construction of Ageing

Within the disciplines of anthropology and sociology, there is an extensive literature concerning the cultural context of ageing and how this informs the ways in which older people feel about themselves [6,7,8,9]. In recent years there has also been a rise in the literature on ‘narrative gerontology’ that seeks to highlight the emic (subjective) perspectives of ageing [10]. While these insights into ‘situated ageing’ can help us to understand the role of culture and the social and historical location of the individual in the process of identity formation [11] the work of Laz [12] helps us to see ageing not as an single construct of ‘being old’ but as an on-going process of adaption. This dynamic perspective is important because older people living with long term conditions and/or chronic pain may have to adapt to a changing physiology and a changing social role.

As Laz suggests, that because we live in biologically ageing bodies, we cannot stop ‘doing’ ageing because it is part of the ‘persistent landscape of the self’. Because ageing is an on-going and interactive process, our expectations of our biologically changing and ageing bodies are continually involved in a process of construction and deconstruction and it is this process that informs our expectations of our bodies. Therefore, although ageing is a biological process, it is through the lens of culture that we give *meaning* to our experiences of ageing. Indeed, as Clark and Anderson [13] have suggested, ageing is ‘a situated phenomena’ and the cultural and historical context of our lives informs the way that we give meaning to the ageing process [14].

### 2.3. Constructing Age Stages

Phases of the life cycle are culturally constructed (e.g., childhood, adolescence, middle aged, old) and they are often presented as a discrete phases of life. For example, Hunt [15] observes that in Western societies notions of a ‘mid-life crisis’ to categorise a period of difficult physical and psychological transition between the ages of approximately 40 and 60 were practically non-existent historically. Cross cultural and anthropological studies confirm that contemporary Western perspectives on mid-life is distinctly unique as ‘in many non-Western cultures, ageing is connected with life experience, wisdom, and thus a positive social status, rather than some form of ‘crisis’’ [16]. Canadian psychologist and psychiatrist Elliot Jaques [17] is credited with coining the term in 1965 after which its usage increased and had become socially legitimised by the mid-1980s to symbolise the arrival of midlife as a precursor to old age and physical and mental decay. Gergen argues that terms like ‘midlife crisis’ and ‘male menopause’ are ‘deficit terms’ that serve as ‘cultural models’, entering the popular vernacular and shaping the construction of everyday reality. As such terms are legitimised by biomedical discourses they ‘increasingly infiltrate everyday intelligibilities [so that the] world becomes increasingly framed by a sense of deficit’ [18]. Gulette [19] and Hepworth and Featherstone [20] argue that these ‘discourses of decline’ represent a convergence between the female and male menopause as both are defined by a desire for youth, physical decay and the potential for everybody to counteract this process through a proliferation of remedies, medical procedures, health and fitness regimes and chemical/hormonal solutions, all of which have become major growth industries in contemporary consumer societies.

In contemporary Western culture, there is a pervasive ideology of ‘active ageing’ that focuses on the productive potential of older people, with a focus on maintaining the body and social activity and living independently [21]. The past 20 years have also seen increasing pressure on workers to remain in the labour force for longer for example, in the UK, the state pension age has risen for future retirees and retirement ages have been scrapped. Macnicol argues that a neoliberal agenda approach to ageing based on individual responsibility has ‘borrowed’ the language of the progressive left, i.e., promoting the removal of barriers to work in later life as an issue relating to inter-generational equity and age discrimination. Consequently, while certain rights and entitlements have been enhanced (e.g., to continue working), other rights have been reduced or removed (e.g., the state pension entitlements, fixed retirement ages). Therefore, as Macnicol, [22] suggests, ‘aspects of the ageing debate have been framed in accordance with neoliberal principles and certain solutions have been presented as natural or inevitable’. Certainly the discourse of, ‘active ageing’ frequently locates the responsibility of physical and mental well-being in later life with the individual. While this ideology can be useful for ‘challenging views of older age as characterised by passivity and dependency’ [23], other authors have expressed concern that it is developing into a moral discourse of responsibility, even though many older people, especially those living with a disability, or are too poor to buy into ‘healthy lifestyle choices’, will not be able to achieve active ageing and longer working lives [24].

So what might be some of the implications of the ‘active ageing’ ideology for older people living with chronic pain? A number of authors have suggested the ideology of ‘active ageing’ can be helpful in challenging ageist stereotypes in wider society [25], yet as other writers have suggested, for many older people, especially those who are disabled, chronically sick or living with chronic pain, these discourses, with an emphasis on a vital, youthful and healthy ageing, hold out an ideal of ageing that will be unattainable [26]. Moreover, Holstein and Minkler have suggested that this has simply created a new ageism that ‘replaces an earlier generalized dread of ageing with a more specific fear of ageing with a disability’ [27].

### 2.4. The Social Determinants of Inequality in Ageing

As suggested above, the cultural construction of ageing can be a useful theoretical and analytical tool as it can help to highlight the temporality and contextual issues of ageing. However, if we merely focus on social constructivist narratives, there is a risk of neglecting the social and political structures that shape the lives and the health of many older people and particularly the lives of older people from BAME communities.

For example, it has been well documented that at a general level, people from ethnic minority communities frequently experience poorer health and more long-term illness, than their White British counterparts [28]. Although there are variations both within and across ethnic groups, research suggests that Gypsies, Irish Travellers, Pakistani, Bangladeshi and Black Caribbean people tend to have poorer health outcomes that all other ethnic groups in the UK [29]. Indeed, life expectancy for adults from Gypsy and Traveller communities has been estimated to be 10 years lower than the national average [30].

A number of factors have been identified to account for these health inequalities across the UK, including racial discrimination [31] and systemic failures in the public sector [32,33]. However, despite the evidence that suggests that people from ethnic minority communities are more likely to experience poorer health outcomes that the majority population, research suggests that they are less likely to access the National Health Service (NHS) [34]. Evidence indicates that many older people from BAME communities are often unaware of public services or how to access them, for example the uptake of end of life care services by members of BAME communities is often low and research suggests that in part, this is due to the fact that palliative care services are often seen as culturally inaccessible [35].

Significantly, these social inequalities seem to persist into old age, potentially reflecting the accumulation of deprivation and discrimination over a person’s life course [36]. However, there remain a number of areas where the evidence base is extremely sparse. The Centre for Ageing Better for example recently stated that there is ‘an astonishing lack of evidence relating to the inequalities in later life experienced by Black and minority ethnic groups, lesbian, gay, bisexual and transgender groups and people with disabilities’ [37]. Similarly there is only very limited research on the impact of migration in later life and clearly there is a need for more comprehensive research in this field.

## 3. Ageing and Pain

As suggested above, culture informs how ageing is constructed in its social context and also shapes the construction and expression of pain; this is because the emotional, verbal and physical expressions of pain are part of a complex interaction between learnt behaviours and biological responses. The following sections highlight a few of the many trans-cultural challenges that health practitioners may face when discussing pain with older people, namely; ageing and pain, the current ideology of ‘active ageing’ and the communication of pain (including different idioms of distress, somatisation and cultural concepts of distress).

### 3.1. Ageing, Culture and Pain

In many cultures, pain is often considered to be a ‘natural’ part of the ageing process [38,39] and many people expect to experience an increase pain as they age [40]. For example, Anderson’s study in rural Nepal found that older people frequently expressed that they had back pain. However, even when medical facilities were made available to them virtually no one attended the clinic. On further investigation, it was found that back pain in Nepalese culture was seen as simply a natural part of ageing.

However, while peoples’ perceptions of ageing and pain are shaped by their culture, thinking about pain and ageing also requires a consideration of some of the deeper, existential questions about our expectations and treatment of older people in our societies. In his influential book, *Being Mortal: Medicine and What Matters in the End*, the surgeon and public health analyst Atul Gawande, examines some of the challenges that health professionals face when dealing with some of complexities of working with older people.

Gawande highlights how medical professionals are trained to respond to pain and suggests that one of the tensions in gerontology is that many professionals have medicalised old age to such an extent, that they no longer accept that ‘life isn’t curable’ [41]. Indeed, while the alleviation and treatment of pain may be considered a moral and professional imperative for health professionals, as Puchalski reminds us [42], ‘compassionate care calls physicians to walk with people in the midst of their pain, to be partners with patients rather than experts dictating information to them’ and the following sections of this paper seeks to examine some of these issues in more detail as we consider some of the dynamics of communicating pain across cultures.

### 3.2. Culture and Different Idioms of Distress

Our culture influences not only how we consider the process of ageing but also how illness and pain are constructed, experienced and communicated [43,44]. Sometimes people do not describe their experiences of pain and ill health in a manner that is recognisable for the health practitioner because patients may use different ‘idioms of distress’. For the clinician, this can be problematic because their patients may not be expressing themselves through a cultural framework that has any meaning within biomedicine [45]. The following two short case studies illustrate the importance of the need for health practitioners to recognise that people often use different idioms of distress to express pain and distress.

#### 3.2.1. Sinking Heart

Krause [46] conducted a study with Punjabis communities living in Bedford (in the UK) and describes how people often express having a ‘sinking heart’. Krause suggests that this term is often used to describe the physical sensations that are experienced in the heart region but it also describes psychological distress, tied to feelings about longing and absent family members. These symptoms are further described through a linguistic framework that draws on language concerning excessive heat, exhaustion, worry and social failure. Krause suggests that these ‘symptoms of distress; are consistent with traditional Indian Ayurvedic medicine but these terms may not be familiar to Western trained medics. Krause suggests that the ‘sinking heart’ can be aligned to a Western model of stress but that the similarity between these two models is in the form, rather than in the content.

#### 3.2.2. Personal and Social Loss

Another example of how different idioms of distress can influence both the clinical encounter and the process of diagnosis is a study conducted by Keys, Kaiser and Kohrt et al. [47]. This research was conducted in Haiti following the 2010 earthquake (and the cultural context of this study is important) and the researchers collected data based on a combination of 11 focus groups, 31 face-to-face interviews and 142 observations of patient-clinician contacts. The research identified 17 different ‘idioms of distress’ pertaining to emotional, cognitive, and psychosocial distress. Their analysis of the data identified that over half of the idioms of distress expressed during clinical consultations related to communication concerning the tèt (head) or kè (the heart). However, in the clinical setting, the health workers only treated the physical interpretation of these expressions of pain and they neglected to have any discussion concerning the emotional and physical context of people’s lives in post-earthquake Haiti (i.e., the loss of friends and family, as well as material and economic resources).

Working with older (and younger) people from cultures that are different from our own, can also present the additional challenge of working with somatic symptoms and in the following sections we shall take a brief look at somatisation and the relationship between this and cultural concepts of distress, both of which, have implications for the clinical encounter when diagnosing pain.

#### 3.2.3. Somatisation

Somatic symptoms sometimes serve as idioms of distress in that somatisation is often defined as a process whereby people express and experience their emotional and psychological distress in terms of physical symptoms and pain [48]. Although a number of authors have also highlighted that somatoform disorders can also be characterized by physical symptoms, for which there are no demonstrable organic causes [49,50,51,52]. Much of the literature on somatisation, suggest that depression and depressive symptoms, are commonly the underlying feature of somatoform disorders, [53]. Traditionally, somatisation in older people (and particularly older women) has been associated with underlying depression [54]. This finding has been supported by more recent research based on a large-scale European study that looked at the prevalence and correlates of somatoform disorders in the elderly [55].

Research also suggests that somatisation is more commonly associated with some cultural groups than others. For example, a number of studies have highlighted how Chinese people are more likely than Europeans to express depression in physical rather than psychological or emotional terms [56]. In trying to account for this variation, researchers have suggested that this may be due to the fact that within Chinese culture, mental illness is often constructed as a moral weakness in the character of individuals. Consequently mental illness is often stigmatised [57,58] and the condition can bring shame upon a family [59]. However, this calls into question when the presentation of distress or pain may be considered as somatisation (i.e., an expression of physical pain with no organic origin), or an idiom of distress (i.e., how peoples’ cultural and personal meaning systems, inform both their health beliefs, that may or may not have an underlying organic origin).

### 3.3. Culture Bound Syndromes/Cultural Concepts of Distress

The labelling of ‘culture bound syndromes’ dates back to the 1960s [60] when the term was often used to describe a combination of psychiatric and somatic symptoms that were considered to be specific to a particular society or culture [61]. Historically, ‘cultural concepts of distress’ were originally constructed as only occurring in rare and exotic instances [62,63] but more recently some authors have critiqued the labelling of ‘exotic syndromes’ by Western theorists who attempted to locate ‘ethnic’ conditions as something separate from the ‘normal’ White, Western range of syndromes [64].

More recently, there has been a tendency to use the term ‘cultural concepts of distress’ which has been added to the Diagnostic and Statistical Manual of Mental Disorders series, with the publication of DSM-5 [65] and it continues to be discussed in the transcultural psychiatry literature [66]. However, as Ventriglio, Ayonrinde and Bhugra have suggested, the rise of discourses concerning ‘cultural concepts of distress’, may actually say more about the rise of Western diagnostic and classificatory systems than to specific health conditions [67]. Moreover, critics such as Obeyesekere [68] have suggested, there are many Western clinical concepts that could be considered as examples of culture-bound syndromes, if viewed form a different cultural lens.

Moreover, as Nichter suggests patients may often wish to share pain or other forms of distress but they can only express this through a known or culturally acceptable disorder [69]. An example of this would be the work of Hinton, Ba and Peou et al. [70], who describes how many Khmer refugees in Cambodia experienced ‘*khya*’ attacks during the Pol Pot period (1975–1979). The authors explain that this is an experience of anxiety where patients fear death due to bodily dysfunction and often describe pain, neck restrictions, digestive problems and dizziness. The condition appears to occur without any external trigger. The authors describe how they tried to deconstruct this cultural concept of distress into a framework of understanding that had meaning for them. Namely, the researchers understood *khya* as being a response to the refugee’s past experiences of violence under the horrific Pol Pot regime and that patients’ autonomic nervous system were often triggered by multiple cues that precipitated the other symptoms. Significantly, the researchers suggest that this was linked to the fact that the wider society was dealing with catastrophic events and that the patients were drawing on culturally known meaning-systems to explain these new embodied experiences. While the example Khmer refugees outlined above may seem far removed from everyday practice working with older people in the clinical setting, what it may help to illuminate is how the social and political context of the patients’ health can result in very different manifestations and expressions of pain and distress. For health practitioners, it is also useful to recognise the inter-subject nature of communication in these clinical encounters and we shall look at this in more detail in the next section.

### 3.4. Inter-Subjectivity in the Clinical Encounter

As suggested above, when health professionals and patients meet, there are always two subjective positions and this is important to remember even if people come from different, similar, or the same cultural backgrounds, we may not always share the same world-views. Csordas has suggested that the skills required for these rich intercultural encounters is ‘attention’ and recommends that what is required is, not just attention to the body, but ‘to the bodies situation in the world’ [71]. Indeed this is an extension of the argument made above that suggests that older people construct their ‘ageing identities’ in the process of interacting with the specific cultural and historic context of their lives.

Nichter extending the ideas of the ‘situated body’ highlights how the clinical encounter is commonly assumed to be a dialogue between two people (the professional and the patient) [72]. He suggests, that in practice, the patient nearly always belongs to wider networks of understanding and therefore Nichter argues, to some extent, the practitioner is always in dialogue with both the individual and the cultural context of their lives (as indeed the case studies in Haiti and Cambodia outlined above). Therefore, when applying these concepts to the clinical encounters with older people expressing pain, practitioners may want to include discussions concerning the wider social context of people lives (such as the role of family, religious beliefs etc.). However, Nichter also offers a note of caution and highlights how some patients may use biomedical terminology (especially when patients can look up health conditions on the internet) and they may suggest to the practitioner that they are suffering from a disorder ‘associated with a biomedical disease category that has or has not been diagnosed by a practitioner’ Yet, as Nichter argues, health professionals need to be aware that the patient may be using a biomedical disease category as an idiom of distress, in order to communicate a different concern. Likewise, practitioners need to be sensitive to culturally shaped taboos in communication. For example Gypsy and Traveller communities often make use of avoidance strategies, such as not acknowledging conditions (such as cancer) or using euphemisms to describe serious conditions, and these strategies are widely used among various social and ethnic groups [73].

So in terms of paying ‘attention’ to pain narratives in older people and listening for different idioms of distress practitioners need to be cautious, not only when they are clearly working across cultures, for example with a Japanese older man and a young Irish nurse but also in working with near-similar cultures such as a young Irish nurse and an older Irish man. This is because even when sharing the same biomedical language and assumed culture, health practitioners and older patients may not be sharing the meaning, even if they are sharing the same language and culture. The role of the professional in such contexts is to be attentive to variability in the expression of distress and the multiple meanings of health that are culturally, historically and personally constructed [74].

## 4. Stoicism and Coping with Pain

The final section of this paper concerns the role of stoicism in culture and the expression of pain. Stoicism is usually described as reluctance to verbally express pain or distress [75] and is commonly linked to culture. The philosophical origins of stoicism date back to ancient Greece, where emotional constraint and fortitude were often promoted as positive qualities of character and strength [76]. However, in the contemporary setting stoicism is also linked to a number of other intersecting factors, including age, class, cultural and religious beliefs, professional training and gender socialisation and these factors often influence the ways in which older people may express and cope with pain.

### 4.1. Generational Stoicism

A number of authors have suggested that older people tend to be more stoic than younger people [77,78], although critiques of age-related stoicism theories have suggested that certain groups of people have often needed to develop stoic attitudes in order to survive, due to the social context of their lives [79]. For example, many older people in the UK lived through the Second World War and they often had little choice but to be stoical. This generation of older people, will have (by necessity), experienced early socialisation into stoicism due to bombings, loss of family, friends and property, shortages of food and other supplies. Stoicism was also seen as part of the national character in wartime and existed as propaganda when national and personal resilience became valorised [80]. Many older people continue to hold onto the idea of being stoic as a positive trait and that complaining may be considered to be a sign of moral and personal weakness and some authors have suggested that this maybe understood as ‘generational stoicism’ [81] and clearly this may inhibit some older people from asking for support in the management of pain.

### 4.2. Stoicism and the Professional Control of Pain

However, it is notable that stoicism is not unique to specific generations and training in the endurance of pain is also a common principle found in some specific professions. Indeed, the endurance of pain is often part of training and constructed as a facet of becoming ‘a professional’. For example, professional ballet dancers [82], footballers [83], boxers [84] and people employed in the armed forces [85] are all expected to endure pain without expressing emotion as part of their professional roles. Of course, for some older people it is possible that this may leave a legacy in their older age, which may inform their modes of expressing and enduring pain, although to date, there has not been any research looking at the dynamics of professional pain endurance and pain management in old age.

### 4.3. Religion, Spirituality and Stoicism

An individual’s spirituality and/or religious beliefs may contribute towards an understanding of the psychosocial factors that inform how older people cope with, and express their pain. A number of studies have highlighted the role of spirituality and religious beliefs in positive mental health outcomes [86,87]. Some authors have highlighted how stoicism is often part of religious and spiritual practice and the enduring of suffering is a commonly held belief in many religious traditions, including Confucianism, Sikhism, Daoism, Hinduism and Buddhism [88]. However, Oman and Thoresen [89] have suggested that the role of spirituality and religious beliefs and its contribution to health can be linked to enhanced social support and the maintenance of positive psychological states, suggesting that this may help to support psycho-neuro-immunological pathways.

However, beliefs about pain and suffering are often central to the wider philosophy of the role of the human body in spiritual life. For example, in Sikhism, someone may refuse pain control because of a belief in *karma* (i.e., a belief that actions over the lifetime of person will informs and change the quality of a rebirth or future life). Consequently, such religions may promote an ideology of stoicism because of the link to moral and spiritual benefit. Although clearly, individuals who follow the Sikh religion will each have their own approaches to pain and practitioners cannot assume that just because an older person is following a specific spiritual or religious path that they do not want, or need pain relief.

### 4.4. Stoicism, Culture and Professional Values

Research suggests that it is not only the beliefs and values of the patient that matters in pain management but that the cultural, spiritual and religious beliefs of health practitioners can also influence pain management for older people. For example, Galanti [90] highlighted how Filipino nurses frequently under-medicate patients who were expressing pain, because stoicism is highly valued in Catholic beliefs and Galanti suggests that by limiting pain medication this would offer the patient an opportunity to demonstrate virtue. Lagman, Yoo and Levine et al. have also noted the common use of the concept of “*bahala na*” (never mind what happens) as a way to cope with life challenges in Filipino culture and it is deeply connected to the notion of “it’s in God’s hands” or “leave it to God” [91].

Another example of the impact of culture on professional practice and pain management is the study conducted in 1999, by Mebane, Roy, Otman et al. The study looked physician’s self-reported attitudes toward end-of-life decision-making and treatment preferences. The study involved a questionnaire with 437 physicians (280 White and 157 African-American physicians) in the USA and the findings indicated that ethnic White American doctors were more likely to promote advance directives and to support early end of life discussions, whereas in contrast, African-American doctors tended to request more life-sustaining treatments. The study concluded that ‘while medical training results in a physician socialization process that provides a common knowledge base for physicians to make clinical decisions, physician attitudes and preferences are also guided by social and cultural factors’ [92].

## 5. Critiquing Cultural Perspectives on Ageing and Pain

In this paper the authors have suggested that as healthcare professionals are now working with an increasingly ethnically and culturally diverse spectrum of older patients, an understanding of the cultural construction of ageing and pain can help to enrich the clinical and intercultural encounter between professionals and older patients. However, while the cultural constructionist models are useful, it is also important to caution against essentialising ‘others’ (i.e., implying that specific ethnic groups have innate characteristics) [93,94]. Moreover, just because a member of staff, or an older person shares some of the same characteristics with others, it does not mean that they maintain exactly the same views or beliefs (for example, not all Punjabis people will understand or articulate their health needs through the same framework of meaning). In addition, there is a risk that by using a cultural construction lens to understand the communication of pain in old age, that we my stereotype specific ethnic groups. For example Diver Molassiotis and Weeks [95] observed that social scientists tend to overlook processes of acculturation and change in the minority individual’s relationship to the majority culture and therefore the experiences of older people from BAME communities will vary. For example, Smith and Moreno found a convergence between UK born South Asians and White British attitudes towards palliative and end of life care, while older people born in South Asian communities, often expressed a preference to either return ‘home’ to die, or to be buried in the country of their birth [96]. Moreover, related to this idea that the concepts of ethnicity and culture are dynamic (and not stable signifiers), it is important to reflect that the term ‘ethnicity’ is also methodologically and conceptually problematic. Ethnic categorisations are themselves socially constructed and this risks homogenising people into pre-conceived categories, which can act to minimise some of the differences within and across groups. It is therefore important to recognise that an older persons’ identity involves both self-ascription [97] and it is also externally constructed/imposed and that we are all engaged with the process of meaning-making our identities in everyday life.

Furthermore, as suggested above, many people from BAME communities experience poorer health and more long-term illness, than their White British counterparts across the UK due to social and economic inequalities, however, by over focusing on issues concerning ‘culture and ethnicity’ we may obfuscate the mechanisms that perpetuate social and economic inequalities [98,99].

In conclusion, in this paper the authors have suggested that the expression, causality and meaning given to pain and old age are often informed by the cultural context of the life of the individual. Although discussions concerning pain and the process of diagnosis are based on medical evidence, it is the clinical encounter where the inter-subjective exchange between the older person and the clinicians takes place, and it is during this cultural exchange that the meaning of pain in old age can be shared.
